# A Refractory Case of Rapidly Progressive Glomerulonephritis Due to Microscopic Polyangiitis

**DOI:** 10.7759/cureus.60366

**Published:** 2024-05-15

**Authors:** Virginia L Hoch, Peter Burke, Ajeetpal S Hans

**Affiliations:** 1 Internal Medicine-Pediatrics, Christiana Care Health System, Newark, USA; 2 Nephrology, Christiana Care Health System, Newark, USA; 3 Internal Medicine, Christiana Care Health System, Newark, USA

**Keywords:** end-stage renal failure, renal failure, immunosuppression, vasculitis, microscopic polyangiitis, rapidly progressive glomerulonephritis

## Abstract

A 75-year-old woman, with hypertension and atrial fibrillation but no prior renal history, presented to the hospital for chest discomfort and dyspnea. She was found to be in acute renal failure, with a serum creatinine of 5.1, increased from a baseline of 0.9, and urine analysis revealing proteinuria and hematuria with dysmorphic red blood cells. Subsequent work up was significant for positive perinuclear antineutrophil cytoplasmic antibody (p-ANCA) and myeloperoxidase antibodies. She underwent a renal biopsy, which revealed necrotizing crescents in 12 of 14 glomeruli, and she was diagnosed with rapidly progressive glomerulonephritis due to microscopic polyangiitis. Despite aggressive treatment with plasmapheresis, high-dose prednisone, and rituximab infusions, renal function worsened, and she required initiation of hemodialysis. She was ultimately discharged after a three-week admission, with plans to continue rituximab infusions and three times weekly hemodialysis in the outpatient setting. Due to her poor response to traditional therapies, initiation of a new targeted immunomodulator known as avacopan, a complement 5a receptor antagonist, was considered. Such targeted immunomodulators are also of particular interest as possible ways to reduce the risk of severe infection associated with current broad immunosuppressive modalities. In addition, when used in place of steroids, they reduce the morbidity associated with cumulative glucocorticoid toxicity. For patients with ANCA-associated vasculitis refractory to standard therapies, targeted immunomodulators such as avacopan should be considered as alternative or adjunct therapy.

## Introduction

Rapidly progressive glomerulonephritis (RPGN) is a syndrome characterized by the following: 1) rapid loss of renal function over days to weeks, 2) nephritic findings on urine analysis (e.g., proteinuria, hematuria, dysmorphic red blood cells, and red blood cell casts), and 3) cellular crescent formation in the glomeruli seen on renal biopsy [1]. Patients who present with RPGN are at risk of progressing to end-stage renal disease (ESRD), especially if renal biopsy reveals a high percentage of glomeruli affected. For instance, if less than 50% of glomeruli show crescentic involvement on biopsy, renal recovery is approximately 90% at five-year follow-up, but only 75% for those with more than 50% of glomeruli involved. Furthermore, if more than 50% of glomeruli show global sclerosis, the renal recovery rate drops to less than 25% at a five-year follow-up [2]. Prognosis improves with early initiation of treatment, but this may be delayed while awaiting renal biopsy or other biomarkers confirming a specific underlying diagnosis. Furthermore, even after diagnosis and appropriate treatment initiation, some patients may prove refractory to traditional therapies. For this reason, treatment models continue to evolve as new therapies are investigated, helping to prevent progression to ESRD for those patients with suboptimal responses to initial therapy.

In this article, we report a case of RPGN due to antineutrophil cytoplasmic antibody (ANCA)-associated vasculitis (AAV), which was refractory to initial induction therapy, requiring consideration of novel immunomodulators. This article was previously presented as a poster at the 2024 National American College of Physicians (ACP) Meeting on April 19, 2024.

## Case presentation

A 75-year-old woman, with a history of hypertension and recently diagnosed atrial fibrillation, presented to the emergency department for chest discomfort and dyspnea on exertion. She was found to be in acute renal failure with a creatinine of 5.1 (increased from a baseline of 0.9 three months prior) and blood urea nitrogen (BUN) of 38. Her remaining electrolytes were within normal limits with a potassium of 4.6, chloride of 100, bicarbonate of 27, calcium of 8.8, and magnesium of 2.0. Her anion gap was also within normal limits at 12. A complete blood count was significant for a hemoglobin of 9.0 (improved from prior baseline of 7.5 three months prior) with a mean corpuscular volume (MCV) of 84.8, but white blood cell count and platelets were appropriate at 11 and 332, respectively. Urine analysis revealed 300 protein, with extensive red blood cell casts and dysmorphic red blood cells. Home medications included amlodipine, metoprolol succinate, apixaban, and furosemide, but no known nephrotoxic agents or other recent exposures.

**Figure 1:**
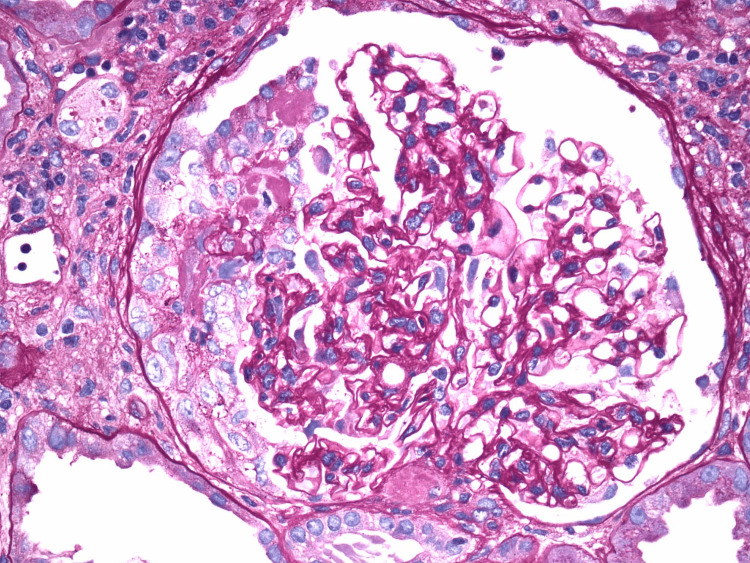
Light microscopy of renal biopsy, showing glomerular crescent formation

Subsequent immunological workup was positive for perinuclear ANCA (p-ANCA) and myeloperoxidase (MPO) antibody and negative for cystoplasmic ANCA (c-ANCA), rheumatoid factor, and double-stranded DNA. Serum C3 and C4 were within normal limits. Renal ultrasound was unremarkable. She underwent renal biopsy, which revealed 12 of 14 glomeruli with necrotizing crescents, but no significant interstitial fibrosis or tubular atrophy (Figure 1). She was diagnosed with RPGN due to microscopic polyangiitis (MPA) in the setting of positive p-ANCA and MPO titers. 

The patient underwent treatment with plasmapheresis and high-dose prednisone and ultimately transitioned to rituximab infusions. Because of a lack of improvement in renal functioning, with creatinine peaking at 9.15 (estimated glomerular filtration rate (eGFR) of 4), she required initiation of hemodialysis. Her hospital course was also complicated by a pleural effusion requiring thoracentesis. Pleural fluid was found to be exudative with cytology negative for malignancy, considered likely to be inflammatory in etiology. After a three-week admission, she was ultimately discharged on a prednisone taper with plans to continue rituximab infusions and three times weekly hemodialysis in the outpatient setting. She was scheduled for a close follow-up with both nephrology, as well as rheumatology with considerations to initiate a new targeted immunomodulator known as avacopan owing to her refractory course.

## Discussion

AAV is an autoimmune disease in which ANCAs affect small blood vessels, causing a necrotizing vasculitis with few or no immune deposits. When the renal vasculature is affected, this can result in hematuria, proteinuria, impaired renal function, and, in severe cases, RPGN. The two major entities causing AAV are granulomatosis with polyangiitis (GPA) and MPA. Immunofluorescence typically reveals perinuclear involvement (p-ANCA) and cytoplasmic involvement (c-ANCA) in MPA and GPA, respectively. GPA commonly affects the kidneys, lungs, and upper respiratory tract, and is characterized by granulomas seen on pathology. It is often associated with proteinase 3 (PR3)-ANCA [3]. MPA lacks granuloma formation and commonly affects the kidneys and lungs but does not typically involve the upper respiratory tract. In approximately 70% of cases, it is associated with MPO-ANCA, but less commonly can be seen with PR3-ANCA [4].

The prognosis for patients diagnosed with AAV is poor without initiation of aggressive immunosuppressive treatment, with a one-year mortality rate of up to 80% [5]. Therapeutic advances over the past 20 years have now improved five-year survival to about 74-91% in GPA and 45-76% for MPA [6]. However, there remains a population of patients for whom the typical treatment pathway is not effective, requiring the consideration of novel therapies.

Treatment for AAV typically involves an induction regimen followed by a maintenance regimen. Patients with severe AAV should undergo induction with glucocorticoids and either rituximab or cyclophosphamide. Plasma exchange may also be considered as well to reduce circulating serum ANCA levels, with recent guidelines recommending seven treatments in 14 days [5]. Induction therapy in non-severe cases involves glucocorticoids in combination with either methotrexate or mycophenolate mofetil [5].

When remission is achieved, maintenance therapy should be initiated to prevent relapse, which occurs in approximately 80-90% of patients who do not receive maintenance therapy [7]. With maintenance therapy, rates of relapse are variable, occurring in 5-50% of patients [7], with risk factors including GPA diagnosis, previous history of relapse, and PR3-ANCA. Common maintenance therapies include methotrexate, azathioprine, rituximab, or mycophenolate mofetil and leflunomide, and treatment duration is typically at least two years [5].

However, these aggressive immunosuppressive regimens put patients at risk for other serious complications, including infection. In fact, several studies have found that infections associated with immunosuppression have become the leading cause of death during the first year of diagnosis with severe AAV, contributing to 34-48% of mortality [8,9]. Additionally, chronic glucocorticoid use is associated with other adverse effects such as weight gain, hypertension, hyperglycemia, hyperlipidemia, steroid-induced myopathy, and skin changes. 

For this reason, more targeted immunosuppressive therapy is under investigation to help reduce toxicity and risk for infection. Avacopan, a complement 5a receptor antagonist and a cytochrome P450 3A4 inhibitor, is one such new drug. It works to block the alternative complement cascade to prevent tissue damage without inhibiting neutrophils [5]. Initial randomized control trials have shown that avacopan alone and in combination with prednisone is noninferior to prednisone alone [10]. Follow-up studies have since shown avacopan to be noninferior and even superior to prednisone at 52 weeks for sustained remission (65.7% in the avacopan arm versus 54.9% in the prednisone arm; p = 0.007 [11]). Additionally, avacopan was found to improve kidney function when compared to prednisone in some subgroups and to reduce overall cumulative glucocorticoid toxicity [11].

## Conclusions

While current immunosuppressive therapies have significantly improved overall survival for patients with AAV, there remains a subset of patients who will prove refractory to these treatments, such as our patient described in the case above. For such patients, targeted immunomodulators such as avacopan may provide a safer and potentially more efficacious treatment for AAV. Further studies are needed to help determine which patients would benefit from the early initiation of novel therapies such as avacopan.
